# Miscellaneous

**Published:** 1857-03

**Authors:** 


### Miscellaneous.

### REMARKS OF DR. E. COLLINS,

[On the first topic of discussion in the Miss. Valley Association.]

In the first place I would state that foil when prepared and used after the following manner is preferable to gold, in any other known form. Fold the foil into strips about one sixteenth of an inch wide; then wind these strips, in a spiral shape, around a small and smoothly polished wire, then gently slide the foil from the wire, at the same time softly twist the free extremity of the gold between the thumb and fore finger. Thus having your gold formed into threads of different sizes nothing now remains for preparing it for use but annealing, or drying which should always be done at the time of using. I lay my gold upon a plate of iron, but never suffer the gold to become red hot, as this hardens it, and renders it unfit for use.

Foil as it comes from the manufacturer is in as malleable state as is desirable. My object in heating my foil is principally to free it from moisture.

My manner of using gold in this manner is as follows: having the cavity perfectly formed, the mouth of the patient dried out as much as practicable, the napkins placed, the patient directed to draw the tongue back against the soft palate to prevent respiration through the mouth, completing the preparatory steps by drying out the cavity, perfectly with dry, warm cotton, I then, with a small serrated pointed plugger, proceed as with crystal gold, commencing at the bottom, or if the bony covering over the pulp is thin, I take the strongest wall of the cavity. If the gold prepared as above, kept perfectly dry, and carefully packed, with the above style of plugger, a better filling is made, than is possible with gold in any other form. In the first place it is better than blocks of foil, being guided and controled by the left hand around the walls of the cavity, followed by the pluggers; its thread-like pliability renders it more susceptible of being compressed into all the irregularities of the walls of the cavity. Should the cavity get wet during the operation, which often happens, notwithstanding the utmost precaution, it can readily be dried again, and the operation proceed, without the least apprehension of its injuring, or rendering the filling imperfect.

In the next place being laminated, it can be more solidly compressed than crystal gold; adhering together by atmospheric pressure, and this more effectually excluding air and moisture than gold in the last mentioned form, which hangs together in a great measure by the interlacing of its crystals, many interstices being left open, through which air and moisture finds ready ingress; especially about frail margins and walls of the cavity when the requisite amount of pressure to consolidate the crystals is the most hazardous and important. In preparing the gold a covering should be worn upon the points of the fingers with which the foil is held to prevent the soiling it by the excretions which are continually passing from them, dryness being indispensable to a perfect adhesion of the gold in this or any other form.

I will venture but a few remarks upon the second topic of discussion.

There are passing out from the pulp chamber a vast number of minute tubes, which are united throughout their whole extent by the interchange of other and smaller tubes. These have their membranous linings, and all their minute ramifications, vessels, and nerves, whose capillaries inosculate at various points, immediately under the enamel. These vessels are different from the arteries and veins only in the color of the fluid they contain, which corresponds with the color of the dentine, as the color of the soft tissues corresponds with the color and quality of the fluids that nourish them. In youth the pulps, the nerves and vessels are correspondingly larger, hence the teeth in early life are more sensitive than in more advanced age, because of the diminution of the pulp and tubuli by ossification. Now taking the above arrangement, it is not unreasonable to conclude that the dentine may become the seat of congestion from obstructed circulation and consequent irritability of nerves without the presence of red corpuscles. In every other respect, inflammation here is identical with inflammation of the pulp of the tooth, they are all incased in bone, so that expansion is impossible, and exalted sensibility is the result.

Upon the other points I have not time to remark at present.

---

QUINCY, Ill., Dec. 20th, 1856.

Tooth within a tooth. Messrs. Editors—It appears to me that the solution of that astonishing anomaly in *Toothology*, or as our friend calls it "freak of erratic nature," is not so very difficult as he seems to suppose.

The pulp and nerves of the tooth had become ossified, and as the pulp cavity resembles in form the outer tooth, of course the denticle removed would if occupying the entire space resemble the tooth from which it was taken.

I have within the last fifteen years met with some three or four cases of ossified pulp. I have now a specimen in my possession taken from a first inferior molar. And one that I removed from the opposite tooth in the same mouth is now in possession of Professor A. Snowden Piggot, of Baltimore. The subject from which they were taken was a young lady not over eighteen years of age.

My partner, Mr. H. N. Lewis, a few months since removed an ossified pulp that had a strongly marked fang.

As a general thing ossific deposits of this kind are granulated, and irregular in form. In cases of exostosis of the fangs we are very likely to meet with ossified pulps.

Hoping I may have in some degree elucidated this matter for your correspondent,

I subscribe myself, Yours truly,

SOCRATES.

---

### DENTAL PERIODICALS.

MESSRS. EDITORS:—In the last number of the Register our remarks on the above subject appears to have been regarded as applicable to the "Dental News Letter." We *cannot* say that we had this exellent periodical in view when we penned the article. We frankly confess, however, since our attention has been turned to it that the criticism is as justly applicable to the "News Letter" as to the Recorder, Forceps or any other publication, not relying on the subscription to the work for support. We wish not to be inviduous and are willing to accord to the News Letter all which its gentlemanly and enterprising proprietors claim and perhaps a little more than they in their modesty might claim. The fact is, since we are forced to think on the subject it is this excellence which makes this periodical appear to have been "referred to." We suppose that gentlemen in the profession who keep "stacks of periodicals" on hand, would have a better estimate of the profession and its literature too, if they had to pay for it and were not made to feel that they were mere advertising affairs.

It is this competition with Journals which can be afforded for nothing, and which are furnished year after year without any expectation to pay—which is; as we think, a just cause of complaint, with those who must look to paying subscribers for compensation.

Hence we asked in our article "would the profession do well and act wisely to rely upon them?" It cannot be supposed that they will be published when the object for which they were started no longer exists.

The "taint of Dental Colleges," is hardly a fair inference, for we presume none of our Colleges rely much upon any particular Dental periodical. For they do their principal advertising through other channels, and perhaps rely as little on the Dental press, as the Medical Colleges do on the Medical. The fact is, our Dental Colleges have enough to do without sustaining Dental periodicals, and it is properly the sphere of the press seeking to elevate the literature of the profession, to aid our Dental institutions in their feeble state all in their power.

Without our Dental institutions, we doubt if a Dental periodical would be sustained at all—our Dental Colleges have become a "fixed fact," and to blot them out, of existence for half a century would be the greatest blow to Dental science which could be inflicted.

We impugn not the motives of any one, and have no doubt, that the motive of the firm which furnishes us with not only good teeth, but a good Dental periodical is a good one; but do object to the unfair competition to which the latter subjects others. We really think that things may become too cheap for the good of the consumer as well as manufacturer. Suppose we should as Dental publishers give every man in the profession what teeth he wanted to use per year to induce him to take our work, would not our worthy friends think the competition rather unfair and troublesome? We believe that the better teeth we manufactured the more it would annoy you, gentlemen, and the less of our teeth could be found in "stacks" to be "overhauled" by the profession. We argue however, that the motive as well the object of the publisher should be canvassed. If the motive and object both are good it is worthy of support. We do not believe it right to "do evil that good may come." It is the motive which stamps crime with its guilt.

The Medical profession would hardly tolerate a Journal the object of which was to advertise the publisher into business. We might cite examples of this kind and which received the general condemnation of the profession.

We admit that the "penny press" has had its use and accomplished its purpose to some extent, and however humiliating it may be, we must confess that there are many in our profession who need *stimulating*, yet we cannot see that they even pay the "penny" for the operation. Hence we so often hear the reply: I would take your paper, but I get the—— and so and so "and they cost me nothing, besides they publish all your best articles."

We will however say to the publishers of the Dental News Letter, that our remarks to which they take exception, were more particularly directed to the profession, hoping to make them feel somewhat the obligations they are under to sustain Dental publications, and that even should they be receiving much which costs them nothing, they are not thus relieved of obligation. Yours truly &c.

JAMES TAYLOR.

---

### ORGANIZATION OF THE ST. LOUIS DENTAL SOCIETY.

At a meeting held December 11th, 1856, at the office of Drs. Dunham and Hale, at which were present, Drs. Dunham, E. Hale, Sr., Isaiah Forbes, H. J. B. McKellops, A. Blake, C. M. Forbes, E. Hale, Jr., Levett, Nichols, Samuels, Hinson, Comstock, Leslie, of the city, and T. Phillips, of La. Dr. Dunham was called to the chair, and A. M. Leslie appointed secretary, pro tem.

It being the unanimous sense of the meeting, that a city society should be organized, a committee was appointed to prepare and present at a subsequent meeting, rules for the government of the society.

At a subsequent meeting, the society was fully organized, by adopting a simplified constitution and by-laws, and the appointment of permanent officers.

The chief features of these laws, are:

"This society shall be called, "The St. Louis Dental Society." "The acting members must be residents of the city or county of St. Louis." "It shall be the duty of the members of this society, (in rotation) to present, at each regular meeting, some case or specimen from their own practice, upon which each member present, is required to present his views."

The officers elect of the Society, are:

*President*, S. Dunham; *Vice President*, A. Blake; *Treasurer*, G. H. Perrine; *Secretary*, A. M. Leslie; *Executive Committee*, H. Barron, I. Forbes, G. H. Perrine.

Since its organization, this society has taken steps well calculated to give permanency to it. A room has been rented and furnished by the association for its convenience. Here, it is hoped it will shortly set up a dental cabinet, which by the liberality of its members and others, will be an object of interest to their brethren visiting the city.

We may here say, that members of the profession passing through the city, are freely invited to its meetings, the members taking pleasure in forming the acquaintance of their brethren from abroad. The regular meetings are held the first Tuesday in each month. Its place of meeting is at No. 79, Market street, over the Dental Depot of A. M. Leslie.

The society has just perfected a fee-bill, which will govern the members in their charges, producing uniformity. It affords the operator liberal reward for his labor, but is not above the rates charged here previously, by the leading operators.

One subject of interest was discussed by the society, at one of its meetings: "What is the best method of folding foil?" proposed by Dr. Isaiah Forbes. His method was to lay two, three, four, five, or six sheets, on the top of each other, and cut them in strips as wide as the length of the cylinder he desired. These strips are rolled on a fine broach, keeping the edges parallel. The varied numbers of sheets give the cylinders different diameters. When preparing a quantity, he puts on fine silk gloves to avoid soiling the gold.

Dr. Spalding also uses cylinders, and folds his foil on a cushion, doubling the leaf on itself, until the thickness and width desired is obtained. He divides the sheets into various sized pieces, by cutting the book and foil together.

Dr. Dunham uses cylinders, and folds on a cushion, which is the general mode of folding among the members.

Dr. I. Forbes, at a subsequent meeting, stated that he had found a great advantage, from placing paper between the shears and foil, when cutting it up. He considered Dr. Spalding had imparted to him a valuable hint.

The subject for discussion at next meeting is, "Is it best to anneal foil after being prepared for the cavity?

Altogether we may say, we anticipate great practical good from this organization.

The members of this society will receive no students for a less term than two years. The preceptor is required to pay a fee into the treasury of the society for each student, which entitles said student to the benefits connected with the society.

Steps have been taken to form a library, for the use of students and others.

The following gentlemen, have signed the constitution:

Isaiah Forbes, C. W. Spalding, Henry Barron, Aaron Blake, S. Dunham, G. H. Perrine, Geo. H. Silvers, C. M. Forbes, I. J. Nichols, F. Hinson, A. M. Leslie, H. J. B. McKellops, David L. Levett, Edward Hale, jr., W. A. Jones, John H. Fairbanks, H. E. Peebles.

On motion of Dr. Perrine,

*Resolved*, The secretary be requested to prepare a synopsis of the minutes of the society for publication.

A. M. LESLIE, Secretary.

### DENTAL SURGERY IN EUROPE.

To the kindness of Mr. Robert LeBlond, whose accuracy and good taste as a proof reader are evidenced by the pages of the "Register," we are indebted for a copy of "The Observer," published in London, dated December, 21st, 1856, from which we make the following extracts:

College of Dentists of England.—A meeting of the members of the Dental Profession. was held on Thursday evening, at the Freemasons' Tavern, Great Queen-street, Lincoln's Inn-fields, for the purpose of forming a college bearing the above title. The chair was taken at 7 o'clock by Mr. Donald Mackenzie, and there were about 200 gentlemen present on the occasion, many of whom came from the most distant parts of the kingdom.

The association, we may here state, was organized by a committee of dental practitioners elected for reporting on the best means of its establishment, at a public meeting of the profession, held in London on September 22d. The report of the committee was presented, received, and adopted at a second public meeting, of the profession held in London on November 11th, on which occasion Mr. Robinson presided, and the committee were re-elected and entrusted with full powers to frame laws and otherwise organize the college.

The Chairman said they had assembled for the purpose of considering the rules framed by their committee for the institution of the society, and afterwards electing their council and officers for the ensuing year. They had undertaken a very difficult task, but he believed that they could by their mingled firmness and moderation surmount all the difficulties which impeded their success, and they would be thus enabled to give to the profession a new dignity and status in the world of art and science.

Mr. Rymer, one of the honorary secretaries, said that at the last meeting a resolution had been passed to the effect that the rules on which the society was to be constituted should be referred to the consideration of a committee. A majority of the members of that committee had thought that they had been empowered to organize the society, and at once frame the laws by which it was to be bound. He had himself differed from that opinion, and he considered that the committee had been invested with no absolute and conclusive authority.

The result of their deliberations was that they had resolved that the rules they had framed should be submitted to the consideration of the present meeting, and should, after the fullest discussion, be open for its sanction or disapproval. Some of the rules had not been brought under the notice of the last meeting, and among the new provisions was one which would exclude from the society any gentleman combining any kind of business with the practice of the dental profession. That rule had been strongly recommended to the committee by six out of the seven dentists practising in Cheltenham; it had also received the approval of a number of other members of the profession; and it had been adopted, after the most mature consideration, by the members of the committee, although they were aware that it would probably give rise to much discussion.

It was then agreed that the rules proposed by the committee for the constitution of the college should be put *seriatim* to the meeting.

The first rule was that the title of the association should be the "College of Dentists of England."

Some gentlemen were of opinion that it would be advisable to extend this appellation, and call the association the "College of Dentists of Great Britain," or the "College of Dentists of Great Britain and Ireland."

It was stated, in reply, that the dentists in Ireland and in Scotland might be anxious to establish colleges of their own, and that it might be considered unfair on the part of that meeting to interfere with their wishes.

The motion that the association should be called the College of Dentists of England," was carried by a considerable majority.

The second rule, defining the objects for which the college was to be established, was adopted without any discussion.

The third rule was to the effect that the college should consist of fellows, members, honorary members, and associates.

A Gentleman wished to know who were the persons that were to be created fellows of the college.

Mr. Rymer said, that the fellows would be the men who had done anything extraordinary for the advancement of dental science.

The fourth rule provided that the college should be conducted by a council of management, consisting of a president, vice-presidents, a secretary or secretaries, and a council of fifteen others; and it defined the mode in which the members of the council should be elected.

The rule was agreed to after a brief discussion; and so were the fifth, sixth, and seventh rules, which provided respectively that the subscription to the college should consist, for a fellow or a member, of a single payment of £21, or an annual subscription of £2 2s.; that every person desirous of being admitted a member should be proposed by two actual members, while the fellows and honorary members should be recommended only by the council; and that the treasurer should receive and keep accounts of all sums due to the college.

The eighth rule was then put. It was as follows:—"No person combining any kind of business with the practice of the dental profession shall be a member of this college."

Mr. HENRY thought that was a most arbitrary rule. Some of the best dentists were also chemists, and he did not see why such men should be excluded from the association.

Mr. CLEMENTS said, he was himself a dentist, but he had as a partner a gentleman who was a chemist, and they were both of opinion that it was advisable to keep the two professions apart [hear, hear]. He wished to know whether a gentleman who had at one time practised as a chemist, but subsequently resolved on confining his labors to dental surgery, would be admissable to the proposed new college.

The CHAIRMAN: We should be most happy to receive him.

Mr. CLARKE, of Richmond, said that he practised both as a chemist and a dentist, and he had qualified himself for acting in the latter character by a regular professional training. He thought it would be most unreasonable to exclude him from the new College of Dentists of England. He had further to observe that he had been invited to attend the first meeting for the formation of the association, and had been assured, after he had stated the two-fold nature of his pursuits, that no objection could exist to his admission to the proposed college.

Mr. RYMER said, that he had certainly informed Mr. Clarke at the commencement of that movement that he saw no objection to the admission of that gentleman to the association; but he had merely expressed upon that occasion what had been his individual opinion.

Mr. CLARKE asked whether it was fair that he should at present be excluded from the association.

The Chairman said he would ask whether it was fair that the junior members of the dental profession, after having duly prepared themselves for its proper practice, should be exposed to a competition with men carrying on some other business with one hand, and taking from them their bread with the other [hear, hear]?

Mr. Clarke said he practised in Richmond, which could not support a mere dentist.

Mr. Gould also spoke against the rule.

Mr. Charles Rogers, said that the College of Surgeons did not prevent their members from practising as chemists. He moved that the rule be expunged.

The rule was carried by a very large majority.

The remainder of the rules were then adopted, the only one which gave rise to any considerable discussion being the eleventh, which provided that no person allowing his name to be associated with unprofessional advertisements should be a member of the college.

One gentleman wished that this provision should be made more stringent by comprising dentists who advertised in any way, and one or two other gentlemen proposed that the rule should extend to all dentists exhibiting show-cases. Neither of these amendments were pressed for the adoption of the meeting.

Mr. Hill, corresponding secretary, explained that the council would interpret the laws for the guidance of the members, and that it was reflecting upon the judgment of those gentlemen to enter into a lengthened discussion upon the matter. The object was to purify the profession; and though the process might prove sharp to some, the end in view would fully justify the free use of the incising knife and cautery.

The following gentlemen were proposed by the committee as the officers of the association, and were elected on a ballot by 52 votes, while only twelve votes were given in favor of the different amendments which it was sought to introduce into the list:

President.—Mr. James Robinson.

Vice-Presidents.—Messrs. Andrew Clark, Samuel Corbett (Dublin), J. S. Imlack (Edinburgh), Norman King (Exeter), James Louis Oxley (Leeds), Robert Reid (Edinburgh), Somerset Tibbs (Cheltenham), F. H. Thompson (Glasgow).

Council.—Messrs. Wm. Crampton W. Hunt (Yeovil), Robert Hepburn, James Harley, W. L. Canton, Henry L. Jacob (Bridgewater), H. Lintott, Donaldson Mackenzie, James Merryweather, — Mitchell, J. B. Murphy (Derby), W. Perkins, Charles Rogers, Adam Thomson, and T. Underwood.

TREASURER.—Mr. Peter Matthews.

HONORARY SECRETARIES.—Messrs. Charles James Fox, Alfred Hill, and Samuel Lee Rymer.

The proceedings terminated at half-past eleven o'clock, with a vote of thanks to the chairman.

To give additional light on the subject of the college, as well as the general state of the profession, we also copy an editorial from the same paper:

THE COLLEGE OF DENTISTS.—The general meeting of dentists on Tuesday evening has had a most satisfactory result—one, indeed, upon which we congratulate the public and the dental profession. There appears to have been, on the part of the leaders of this movement, a confidence in themselves as well as in the justice of the cause they had undertaken, which cannot be too highly commended. From what has transpired at the public meetings, and in letters addressed to the medical journals, it would appear that eighteen members of the College of Surgeons practising as dentists took upon themselves *secretly* to memorialize their college to grant dental diplomas without consulting the whole body of dentists. No sooner was this discovery made known amongst the dentists generally, than feelings of indignation were aroused, and the *secret* proceeding of the memorialists was publicly condemned. Many of the leading practical dentists immediately put themselves at the head of the movement, with a determination to oppose any interference on the part of the College of Surgeons, the council of which has very properly treated the memorialists' invidious petition with silence, if not contempt, the result probably of the secret manner in which the attempt was made to injure their fellow-practitioners. In a letter addressed to the *Medical Circular*, the writer, a member of the College of Surgeons, observes, when referring to the memorialists, who stated—

"In accordance with the *general feeling of the profession that a Society of Dentists should be formed;* for which purpose a meeting was convened in November, 1855, and at which meeting it was decided to memorialize the College of Surgeons. Now, I ask any one of ordinary capacity whether, on seeing the above statement, the existence of a *general feeling* in a large body of men, urged as a plea, for pressing the subject on the notice of the authorities, would not suppose that the body itself, or its delegates, had been *called together* to give it utterance? Of course he would. No such thing; on the contrary, the body, with its general feeling, knew nothing of what some of the fingers and toes had done until many months after the memorial had been presented; that they had no share in getting it up will be clear to any one who will take the trouble to read the list of eighteen names appended to it, and unless the said eighteen embody in themselves the *general feeling* of the profession, the pretense set forth in the prospectus is a false one. We are further informed on the one hand that the memorial presented to the college twelve months since, is still under the consideration of the council, and on the other it is asserted that the college at once told the memorialists that their charter gave them no power to comply with the request; but if the college had the power it would do so. Would it? Then why has it remained twelve months under consideration? Many hard things are said of the College, and perhaps she has not been a kind mother to all her progeny; but I can never think so badly of her as to believe that, while in all other departments the standard of qualification is being raised, she would take a step, or rather a stride, backward, to suit the convenience of *individuals*, and to perpetuate an evil which ought to be remedied at the very earliest moment."

The authorities from this have taken no notice, and it is to be hoped, in their capacity as a public body, they will not commit such an act of invidious partiality. At a former public meeting of dentists, what was the general feeling on that occasion? One speaker observed, "He did not agree with the eighteen gentlemen who had taken upon themselves the grave responsibility of memorializing the college to grant dental diplomas without first consulting their entire body. He considered they were quite competent to find examiners amongst themselves, and required no reflected light from a foreign college. They were an independent body of practitioners, and as much as he respected the College of Surgeons, and their examination upon surgery, they were totally unqualified to examine upon dentistry. Moreover, he remarked, when was any member of the college who practised as a dentist ever invited to take a seat at the council board? a somewhat significant hint to the profession generally, that our calling, as a scientific body, could not be raised by any alliance." Another, when referring to this matter, observed, "Much as he revered the god-like profession of medicine and surgery, and much as he esteemed those men who were its brightest ornaments, yet he felt convinced not one was qualified to examine upon the science of dentistry; and he was moreover certain they themselves would be the first to admit this fact. Suppose the College of Surgeons had entertained such a monstrous proposition as that of these memorialists, what would have been the *status* of the many hundred practitioners, not members of the college, many of whom are the leading practical dentists of this country? Had the council of the college attempted legislation upon such a subject they would have committed an act of public injustice, and would have arrogated to themselves a knowledge of a science of which they are professedly ignorant; they would have deteriorated their own respected position, and it might have been said *truckled* to a few persons, who, in themselves form an insignificant number of the dentists of these kingdoms. If these persons, however were so anxious to raise the character and *status* of their calling, as their memorial professed, why did they not come forward and assist in forming the College of Dentists at the recent meeting?"

The committee, who have hitherto had the management and arrangement in forming the intended college, have acted most judiciously in holding their meetings publicly, and in admitting amongst their members the assistants as their associates. By this liberal course of action, suspicion has been disarmed, and at a recent public meeting of dentists and their assistants, held at Cheltenham, presided over by the chief practitioner of the town—with one exception—the entire body sent in their adhesion to the college. Members who have enrolled their names, although as yet not made public, will shortly be found in all the provincial towns in England, and before long there will probably be no dental practitioner of any *standing* but will become or should be a member of the dental college. By these means the public will be able to discover in a short time to whom they can apply for dental assistance with safety, and dental quackery and extortion will be a thing of the past.

The general laws adopted at the public meeting, will shortly be published, the first meeting of the council being summoned for to-morrow (Monday).

